# Mr. Verity's Amalgamation Scheme

**Published:** 1920-11-20

**Authors:** 


### MR. VERITY'S AMALGAMATION SCHEME.

Notoriously Charing Cross and at least one other London hospital have come through the bad times with far less of the financial nip than some other sister institutions. Their own outlook on the situation must be different, and the public outlook, in view of their successful struggle, must be modified. We think but few belong to the school of thought of those who are ready to hand over the voluntary system to the London County Council or any other health authority. Part of our large artillery has not yet been fired, and the fighting so far is but in the front trenches. Mr. George Verity, the Chairman of Charing Cross Hospital, is the man at the post of command who has, in not too promising times, dug his institution out of a rut of debt and placed it upon firm financial ground. He believes in amalgamation and co-ordination; he thinks the public can still find the money; he would give statutory powers to the King's Fund. At Charing Cross, Mr. Verity points out, the public have paid off a debt of £100,000, with the result that the whole hospital is open, full from attic to basement, beautifully equipped, and free from all money worry.

He considered that half the deficit on London hospitals could be met by amalgamation, combined buying, interworking, and postponement of ill-digested development schemes. The remainder could be met by payment from patients. No Government interference is wanted, but statutory powers to compel amalgamation and economies should be conferred on the King Edward's Hospital Fund authorities. Adjoining Charing Cross is the Westminster Ophthalmic Hospital, an excellent institution, which apparently Mr. Verity thinks would be still more excellent if a door were knocked through the party wall. We like Mr. Verity's cheeriness, but we do not agree with extension of the powers of King Edward's Hospital Fund as at present constituted. We quite see that necessarily in front of his outlook lies a splendid institution which has emerged triumphantly from crushing financial troubles, and another satisfactory hospital whose geographical position must give rise to certain ideas. But Mr. Verity's theme is in a key of courage and hope, and should inspire us in the midst of the sombre counsels that come from other quarters.

